# Self-Collected Specimens Revealed a Higher Vaccine- and Non-Vaccine-Type Human Papillomavirus Prevalences in a Cross-Sectional Study in Akuse

**DOI:** 10.1155/2020/8343169

**Published:** 2020-01-22

**Authors:** Adolf K. Awua, Alberto Severini, Edwin K. Wiredu, Edwin A. Afari, Vanessa A. Zubach, Richard M. K. Adanu

**Affiliations:** ^1^Department of Epidemiology and Disease Control, School of Public Health, University of Ghana, Accra, Ghana; ^2^Cellular and Clinical Research Centre, Radiological and Medical Sciences Research Institute, GAEC, Accra, Ghana; ^3^National Microbiology Laboratory, Public Health Agency of Canada, Winnipeg, Manitoba, Canada; ^4^University of Manitoba, Winnipeg, Manitoba, Canada; ^5^Department of Pathology, School of Biomedical and Allied Health Science, University of Ghana, Korle-Bu, Ghana; ^6^Population, Family and Reproductive Health, School of Public Health, University of Ghana, Accra, Ghana

## Abstract

**Background:**

Population-specific epidemiologic data on human Papillomavirus infection, which are limited in most of the SubSaharan African countries, are necessary for effective cervical cancer prevention. This study aimed to generate population-specific data on human Papillomavirus infections, and determine which of these, self-collected and provider-collected specimens, gives a higher estimate of the prevalence of human Papillomaviruses, including vaccine and non-vaccine-type human Papillomavirus.

**Methods:**

In this cross-sectional study, following a questionnaire-based collection of epidemiological data, self-, and provider-collected specimens, obtained from women 15−65 years of age, were analysed for human Papillomavirus types by a nested-multiplex polymerase chain reaction, and for cervical lesions by Pap testing. HPV data were categorised according to risk type and vaccine types for further analysis.

**Results:**

The difference between the overall human Papillomavirus infection prevalences obtained with the self-collected specimens, 43.1% (95% CI of 38.0–51.0%) and that with the provider-collected samples, 23.3% (95% CI of 19.0–31.0%) were significant (*p* ≤ 0.001). The prevalence of quadrivalent vaccine-type human Papillomaviruses was 12.3% with self-collected specimens, but 6.0% with provider-collected specimens. For the nonavalent vaccine-types, the prevalences were 26.6% and 16.7% respectively. There were multiple infections involving both vaccine-preventable and nonvaccine preventable high-risk human Papillomavirus genotypes.

**Conclusion:**

The Akuse subdistrict can, therefore, be said to have a high burden of human Papillomavirus infections, which included nonvaccine types, as detected with both self-collected and provider-collected specimens. These imply that self-collection is to be given a higher consideration as a means for a population-based high-risk human Papillomavirus infections burdens assessment/screening. Additionally, even with a successful implementation of the HPV vaccination, if introduced in Ghana, there is still the need to continue with the screening of women.

## 1. Introduction

Of over 200 genotypes of the ubiquitous human Papillomaviruses (HPVs), about 50 are known to infect the mucosal epithelia of the following parts of the human body; cervix, vagina, vulva, penis, anus, and the oropharynx [1–3], often with only a small fraction (about 10%) of these infections persistent and progress to malignancy [4]. These viruses reach these sites by different forms of sexual contacts, including penetrative vaginal, anal and oral sexual intercourse, genital contacts, and genital skin to skin contact, as well as by insertion of unclean foreign objects/substance and by self-inoculation [5, 6]. The leading HPV related cancer is the cancer of the uteri cervix, which is the second commonest cancer among women globally. The global estimated burdens of cervical cancer, in the year 2018, were 570,000 incident cases, and 311,000 deaths [7]. The estimated burdens of cervical cancer in Ghana was 3,151 incident cases and 2,119 deaths in the year 2018 [8]. The crude incidence rate of cervical cancer in Ghana has been estimated at 24.3 per 100,000 population per year [9]. These and other publications clearly aver the fact that HPV infections and cervical cancer continue to be a significant global and national public health concern [10, 11].

The epidemiologic data on the human Papillomavirus infection, and related cancers and precancers have been shown to vary between geographical areas of the world [12] and to be very important for the prevention of the same. For instance, the prevalence of HPV among women with normal cytology ranges between 1.4% in Europe and 24.6% or higher in Africa [12, 13]. Therefore, each population needs to tailor its preventive efforts according to its burdens and epidemiologic data. However, the recommendation of WHO is that girls should be vaccinated since there is increasing evidence of its effectiveness at preventing HPV infections and consequentially as a tool for cervical cancer prevention [14].

However, among over 6.5 million women are at risk of cervical cancer in Ghana, data on the prevalence of HPV both in the general population and among women with all grades of cervical lesions are limited. Additionally, in spite of its indicated advantages [15–17], there is yet to be an evaluation of the usefulness/usability of the self-collection method in Ghana. These limited population-level epidemiologic data may be reliably obtained with either self-collected or provider-collected specimens, however, differences have been reported between data obtained with both specimens among the same population of women [18–21].

The few hospital-based studies (which used provider-collected specimens) in Ghana, have reported varying prevalences of HPV infections among different groups of women diagnosed with different stages of cancers and precancers. These include prevalences of 10.7% among women who reported for cervical cancer screening at a tertiary hospital [22]; 98.0% [23], 89.8% [10], and 93.9% [24] all among women diagnosed with high-grade cervical lesion or cancer between the years 2004 and 2010. These are however not enough for the assessment of the population-specific impact, in Ghana, of the available bivalent, quadrivalent, and nonavalent HPV vaccines.

Therefore, both collection methods were employed in this study with the aim of generating baseline, population-based prevaccination HPV infection and cervical lesions prevalences of a purported high-risk community. Specifically, the aim was to estimate the proportions of the overall HPV infections burden that are due to high-risk HPV infections which may or may not be prevented by the available bivalent, quadrivalent, and nonavalent HPV vaccines.

## 2. Materials and Methods

The data presented herein was part of the findings of a larger cross-sectional study, for which most of the methods presented herein have been previously published [25, 26].

### 2.1. Study Location and Sample Size

The Lower Manya Krobo District was selected for this study because it has consistently (since 2010) been the district with the highest prevalence of sexually transmitted infection (STI) in Ghana, and therefore considered a high-risk location for HPV infections. Using random selection, the Akuse subdistrict, which was made up of 18 communities, (centred at the coordinates 6″06′02.43N and 0″7′16.87E) was selected from among the five sub-districts. At the time of the study, the estimated population of women between the ages of 15 and 64 years, was 2595, out of the total multiethnic population of 8887 persons. The sample size for the study was determined with the random sample size equations and a modification (*S*) since the population was less than 10000. The expected HPV prevalence (*p*) and the degree of accuracy (*d*) used were 26.3% and 0.065 respectively (these were based on studies in the neighbouring West African countries), Furthermore, being a cross-sectional study, a design factor of 1.2% was applied and then based on literature, an estimated response rate to cervical screening of 60% was accounted for. The sample size of a minimum of 352 women for the study was thus obtained. The number of women recruited from each community was distributed by probability proportional to the size of the communities.

### 2.2. Study Population

In the cross-sectional study, 473 women were invited, between March 2012 and March 2013, by a house-based survey to participate. Of these women, 415 (87.7% response rate) consented and completed the study questionnaire. Although they were all invited for the collection of specimens, either at the Akuse Government Hospital or at a specified location within their communities [25], a total of 253 women reported to the location and provided either or both self-collected and provider-collected specimens; therefore, the response rate relative to the population and to consenting women were 53.4% and 61.0% respectively. To achieve a wider coverage of houses within each community, not more than 3 women per house were included in this study, if they were between the ages of 15 and 65 years, had ever had penetrative vaginal sexually intercourse and were willing to provide specimen by at least one of the two collection methods. When a house had 2 or 3 households, at least 1 of the 3 women, was selected from each household. However, where there were more than 3 households in a house, 3 households were initially randomly selected, after which a woman was randomly selected from each. The selected women who had their menses were asked to come back one week after their menstrual period had ended. Women who either were pregnant (self-report), had undergone hysterectomy or cervical conisation were excluded from the study. Two trained Public Health Nurses assisted the study participants, by a one-on-one interview, to complete the study questionnaire, which was used to obtain information on socio-demographic characteristics, sexual behaviour, sexual and reproductive history, menstrual characteristics, use of oral contraception, history of sexually transmitted infections, and cervical cancer screening history.

The study was approved by the Ethics Review Board of the Ghana Health Service (ID No GHS-ERC: 06/11/10). Informed consent, and parental consent and child assent (between 15 and 18 years of age) were obtained from or/and for the participants (as it applied) after explaining the main research goals, sample collection procedures, potential benefits and harms, and privacy and confidentiality of data collected.

### 2.3. Collection of Specimens and Smear Preparation

The methods used for the collection of the specimens were as previously reported by Awua et al. [26]. Specifically, each participant was first instructed on how to use the Rover® Viba-Brush vaginal sampler, (Rovers Medical Devices, The Netherlands). The self-collected specimen was transferred into a sterile 15 mL screw-capped tube containing 5 mL of Phosphate-buffered saline (PBS) with 5 mM of Ethylenediaminetetraacetic acid (EDTA), pH 8.0. These specimens were designated as Self-Collected (SC) specimens for HPV testing.

Thereafter, a health personnel then performed a standardized vagina examination and, with the aid of a sterile plastic disposable speculum, and the PAP-PAK® Cytology kit (Medical Packaging Corporation, Camarillo, USA) collected cervical specimens according to the manufacturer's instructions, for Papanicolaou (Pap) smear and HPV testing. Briefly, the head of a wooden Ayre's spatula was used to collect cellular material from the cervix and the squamocolumnar junction, and then a cytobrush was used to collect cellular material from the endocervix. Each of these was smeared uniformly on separate sections of a microscopic slide (for Pap testing). Thereafter, a fixative (95% ethanol and 5% polyethylene glycol), provided with the kit, was applied. The cytobrush was then rinsed in 50 *μ*L of DNAgard®Tissue (Biomatrica, Inc**., **San Diego) in a 2 mL screw-capped microcentrifuge tube and the head of the wooden spatula was broken and added to the same 2 mL screw-capped tube to complete the sample used for HPV testing. These specimens were designated as Provider-Collected (PC) specimens. All specimens for HPV testing were transported at 4°C and store at −20°C until used for HPV testing.

### 2.4. Staining and Cytology

The slides were completely immersed in two changes of 95% ethanol, overnight. Thereafter, they were rinsed with a continuous flow of running water for 5 minutes and then stained with Mayer's Hematoxylin for 5 minutes. The slides were washed in running water for 5 minutes, after which they were dehydrated by immersion in 95% ethanol for 2 minutes. These were passed through Orange G-6 for 2 minutes, followed by two rinses with 95% ethanol, each time for 10 seconds. The slides were then transferred into a solution of EA for 3 minutes and rinsed very well with two changes of 95% ethanol, each time for 10 seconds. Dehydration was completed in absolute ethanol for 1 minute. The slides were air-dried on the bench, cleared in xylene and coverslip fixed with DPX mountant (GCC Diagnostics, Gainland Chemical Co., Sandycroft Flints, UK). The slides were then examined for cellular characteristics by two cytotechnologists using an Olympus BX51TF microscope with an Olympus DP20 camera (Olympus Corporation, Tokyo, Japan). The slides for which there was no consensus between the two cytotechnologists, a cytopathologist examined the slides. Additionally, the cytopathologist examined all slides reported as unsatisfactory and also, 10% of the slides for which there was a consensus between the cytotechnologists. Included in the slides for examination were previously confirmed slides that were positive and negative for cervical lesions.

### 2.5. DNA Extraction

The DNA extraction method described herein was followed as previously described by Awua et al. [26]. Specifically, the volume of each PC specimen was made-up to 1 mL with PBS and vortexed thoroughly. DNA were separately extracted from 200 *µ*L of each specimen (SC and PC) on the MagNA Pure LC automated system (Roche Molecular Systems, Inc, Pleasanton, USA), using the MagNA Pure DNA Extraction Kit (Roche Molecular Systems, Inc, Pleasanton, USA) according to the manufactures specification, but with the following modification; which was the addition of 15 *µ*L of RNase to the lysis buffer per specimen. The negative controls included for each set of extraction were PBS, DNase/RNase free water, and DNAGard, while the positive control was a suspension of HeLa cells with an integrated HPV 18 genome. The specimen/DNA quality was assessed by real-time PCR amplification of the housekeeping gene, RNase H was added in 10 *µ*L of the DNA extract and analysed with a Light Cycler 480 and related software (Roche Molecular Systems, Inc, Pleasanton, USA).

### 2.6. Nested-Multiplex PCR Based Detection of HPV Genotypes

The extracted DNA were amplified by nested PCR reactions using PGMY09/PGMY11 and GP5+/GP6+ primers, as previously described by Zubach et al. [27] and Awua et al. [26]. The typing of 46 mucosal HPV types were carried out by a multiplex system based on the xMAP® technology, as previously described by Zubach et al. [27] and reported by Awua et al. [26]. Briefly, 46 fluorescence sortable microspheres (Luminex Corporation, Austin, TX) were coupled to the 46 specific probes for the HPV types; 6, 11, 13, 16, 18, 26, 30, 31, 32, 33, 34, 35, 39, 40, 42, 43, 44, 45, 51, 52, 53, 54, 56, 58, 59, 61, 62, 66, 67, 68, 69, 70, 71, 72, 73, 74, 81, 82, 83, 84, 85, 86, 87, 89, 90, and 91. The double-stranded second-round PCR products, labelled with biotin, were made single-stranded by digestion with 2 *µ*L of bacteriophage T7 gene6 exonuclease (New England BioLabs, Pickering, ON, Canada) that removed the non-labelled strand after 40 minutes incubation at room temperature. The single-stranded HPV DNA were incubated for hybridization at 60°C for 10 minutes. Streptavidin-phycoerythrin (PE) (Invitrogen) in 1-tetramethyl ammonium chloride (TMAC) (Sigma), was subsequently added and incubated for 5 minutes at 60°C. Genotype specific hybridizations were detected on a Luminex Liquid Chip 200 flow cytometer (Qiagen) using the Luminex IS software (Luminex).

### 2.7. Statistical Analysis

Frequencies, proportions or/and 95% CI of the proportions were used to describe the distributions and summarize data on specimens collected, HPV genotypes detected, HPV infections, high-risk types of infections, low risk types of infections, and vaccine-type HPVs infections (quadrivalent vaccine-type HPVs are HPV 6, 11, 16, and 18; nonavalent vaccine-type HPVs are HPV6, 11, 16, 18, 31, 33, 45, 52 and 58). Chi-square analysis was used to determine the statistical significance of the differences in the overall HPV, high-risk HPVs (16, 18, 31, 33, 35, 39, 45, 51, 52, 56, 58, 59, 68, 73 and 82) and low-risk HPVs (6, 11, 40, 42, 43, 44, 54, 70, 72 81) prevalences between the two collection methods. The Cohen's Kappa analysis was used to determine the extent of agreement between the HPV infections determined with self-collected specimens and those with provider-collected specimens. The McNemar's test was used to determine the statistical significance of the differences between the concordances in the detection of any HPV infection (overall) and high-risk HPV infection using specimens collected by each of the two methods. The associations between the sexual characteristics and HPV positivity for each of the HPV infection categories (overall, high-risk HPV and Multiple HPV infection) were obtained by logistic regression.

## 3. Results

### 3.1. Demographic and Sexual Characteristics

Of the 473 women invited to participate in this study, 415 (87.7%) agreed to and completed the study questionnaire. A total of 253 (60.96%) of 415 women who consented to participate in the study provided specimens. Tables 1 and 2 present a description and a Chi-square comparison of the demographic, and sexual behaviour characteristics of the women who either reported or did not report for specimen collection.

### 3.2. HPV Genotype Specific Prevalence

The quality of each of the 477 specimens obtained from the 253 women who provided specimens for this study was tested by real-time PCR amplification for the housekeeping gene, RNase H. Although 17 of the 244 self-collected and 6 of the 233 provider-collected specimens were not consistently positive when they were each amplified three separate times for the RNase H gene, they were considered appropriate for further analysis. This was because the criteria used for indicating a sample as inappropriate for further analysis was that allthree RNase H amplifications with the sample were negative.

However, only HPV data of the 226 women who provided both SC and PC specimens were used for the comparisons and the analyses of agreement between the two methods, and the determination of associations between participant characteristics and the categories of HPV positivity.

Out of the 46 HPV genotypes tested for, 37 and 26 types were detected with the SC and the PC specimens respectively (Table 3). Overall, 38 genotypes were detected, but 25 genotypes were detected with both the collected specimens. The eight genotypes that were not detected at all were; HPV11, HPV13, HPV26, HPV32, HPV 34, HPV61, HPV71, and HPV89. On the other hand, HPV43 was only detected with PC specimens. However, HPV33, HPV44, HPV53, HPV68, HPV69, HPV70, HPV72, HPV73, HPV85, HPV86, HPV90, and HPV91 were detected with only SC specimens.

The six most prevalent HPV genotypes detected with the SC specimens were HPV16 (5.9%), HPV35 (4.7%), HPV40 (4.7%), HPV45 (4.3%), HPV58 (4.0%), and HPV18 (3.6%). Those detected with the PC specimens were HPV35 (2.8%), HPV58 (2.8%), HPV16 (2.4%), HPV18 (2.4%), HPV66 (2.4%), and HPV45 (2.0%). For each of the detected genotypes, a higher prevalence was obtained with the SC specimens, however, the differences were not significant; details are shown in Table 3.

### 3.3. Vaccine-Types, Risk Types, and Overall HPV Prevalences

It was noted that the vaccine-type, HPV11, was not detected at all among the women in this study (Table 3). Therefore, quadrivalent vaccine-type (QVT) HPVs referred to in all analyses were HPV 6, 16, and 18; these were detected among 31 of the 244 self-collected specimens, resulting in a prevalence of 12.7% (95% CI of 8.95–17.3%). On the other hand, 15 of the 230 provider-collected specimens were positive for quadrivalent vaccine-type HPVs, resulting in a prevalence of 6.5% (95% CI of 3.84–10.3%).

Furthermore, considering the bivalent vaccine-type HPVs (that is HPV 16 and 18), they were detected in 24 (36.4%) of the 66 self-collected specimens, which were positive for high-risk HPV types. Using the provider-collected specimens, the bivalent vaccine-type HPVs (HPV16 and 18) were detected in 12 (31.6%) of the 38 specimens that were positive for high-risk HPV types. Details are provided in Table 4.

Of the 244 self-collected and the 230 provider-collected specimens, 67 and 33 specimens, respectively were positive for nonavalent vaccine-type (NVT) HPVs (HPV 6, 16, 18, 31, 33, 45 52, and 58), additional distributions are shown in Table 4. Furthermore, 60 (90.9%) of the 66 high-risk HPV types detected with the self-collected specimens and 38 (78.9%) of high-risk HPV types detected using the provider-collected specimens were the high-risk types of the nonavalent vaccine-types HPV (HPV 16, 18, 31, 33, 45 52, and 58). The prevalences of high-risk, probable high-risk (PHR), and low-risk HPV types determined with each set of the two specimen types were also presented in Table 4. The overall prevalence of HPV infections as determined with the 244 SC specimens was 43.1% (95% CI 38.0–51.0%), while that determined with the 233 PC specimens was 23.3% (95% CI of 19.0–31.0%). The difference between these prevalences was significant (*χ*^2^ = 28.943*; p* ≤ 0.001; Table 4). The findings for single, multiple, and vaccine-type HPVs infections are shown in Table 4.

### 3.4. Agreement of HPV Positivity with SC and PC Specimens

The Cohen's kappa (*k*) analysis for each of the HPV infection categories (Table 4), using data of the 226 women who provided both SC and PC specimens, showed significant (*p* ≤ 0.001) moderate levels of agreement for high-risk HPV infections (*k* = 0.402), low-risk HPV infections (*k* = 0.328) and overall HPV infections (*k* = 0.321).  Table 5 presents the findings for overall HPV detection concordance and discordance, which were 67.3% (44 + 108 of 226) and 32.8% respectively, as well as those for high-risk and low-risk HPV types, and the McNemar test with *p*-values indicating the concordances was significantly less than 100%.

### 3.5. Sexual Characteristics and HPV Positivity

The crude odds ratios for the associations between some of the sexual characteristics and the HPV infection positivity categories (overall, high-risk HPV and Multiple HPV infection) were detailed in Table 6. Age, current number of male sexual partners, lifetime number of male sexual partners, and history of STI infection were not significantly associated with HPV positivity. AFSI was only significantly associated with high-risk HPV infection positivity by SC specimens, the odds ratio (OR) was 0.46 (95% CI 0.24–0.85).

### 3.6. Cytology/PAP Smear

Of the 251 microscopic slides prepared and evaluated for malignant lesions, 212 (84.4%) had satisfactory smears, while 39 (15.6%) had unsatisfactory smears for evaluation. All the evaluated satisfactory smears (*n* = 212) were negative for cervical malignant lesion and showed no dyskaryosis and intraepithelial lesions. However, 19.5% (*n* = 41) of them were diagnosed with other conditions, the commonest of which was bacterial vaginosis (Table 7).

## 4. Discussion

To assess the potential impact of HPV vaccination on the burden of HPV infections among women living in the Akuse subdistrict in Ghana, this study determined the distributions of 46 of the 54 genital HPV genotypes instead of the usual 37 HPVs [27–30]. This was to ensure that comprehensive prevaccination baseline HPV data are available for the identification of changes in nonaccine-type HPV distribution, taking decision on the choice of vaccine and postvaccination assessments [31]. To the best of our knowledge, this is the first report on self-sample collection for determining HPV prevalence for a Ghanaian population. It should be noted that an earlier publication presented the age distribution of overall and risk-type HPV infections [26]; however, as indicated above, this article presents the detail of the prevalences of the individual genotypes and the related implications for HPV vaccination in Ghana.

The general nonsignificant difference between the women who reported and those who did not report for sample collection (Tables 1 and 2) indicates that the sampling procedure employed was able to avoid selection bias and therefore ensures a high extent of representativeness and generalisability of the findings of this study. An extensive discussion of the high number of women who even though agreed to participate and signed the informed consent but never showed up for sample collection (screening response rate) has been presented in an earlier publication [25]

Based on the HPV data presented (Tables 3 and 4), when an HPV vaccination is conducted in the study district, the extent of the reduction in HPV burden is expected to be high, particularly, based on the data obtained with the self-collected specimens. However, in spite of the potential benefits of vaccine introduction, the data on multiple infections involving both high-risk vaccine-type HPVs and high-risk nonvaccine-type HPVs, (Table 4), imply that there will remain a small risk for the potential development of cervical cancer. The HPV data on self-collected specimens (Table 4) indicated that even after a successful introduction of the bivalent vaccine or the quadrivalent vaccine, the 6.42% and 9.17% multiple infections respectively involving high-risk vaccine-type HPVs and other high-risk vaccine-type HPVs are most likely not going to be completely prevented. On the other hand, based on the HPV data on provider-collected specimens, only a few infections are most likely are not going to be completely prevented, (1.69% multiple infections involving both quadrivalent vaccine-type HPVs and other high-risk vaccine type-HPVs). Furthermore, when a nonavalent HPV vaccine is introduced, the data on self-collected specimens imply that the 11.00% multiple infections involving both high-risk nonavalent vaccine-type HPVs and high-risk nonnonavalent vaccine-type HPVs may not be completely prevented; HPV data on provider-collected specimen imply that the 5.08% multiple infections involving both high-risk nonavalent vaccine-type HPVs and high-risk nonnonavalent vaccine-type HPVs may not be completely prevented. These remaining high-risk HPVs following a quadrivalent vaccine introduction, per data on self-collected specimens, will most likely be HPV59, HPV58, HPV51, HPV 45, and HPV35. Following a nonavalent vaccine introduction, these will most likely be HPV59, HPV51, HPV39, and HPV 35. However, based on the HPV data on provider-collected specimens, the remaining high-risk HPV infections after a successful introduction of quadrivalent or nonavalent vaccines, will both be HPV59 and HPV52. Considering that cross-protection occurs following quadrivalent vaccine introduction (against HPV31, HPV33, and HPV45) [32–37], the remaining HPV burden indicated above, as per HPV data on self-collected specimens, will be HPV59, HPV35, HPV51, and HPV58. As per the data on provider-collected specimens HPV59 and HPV51 most likely will continue among the population. These imply the need for the development of additional HPV vaccines, which will target these four HPVs (HPV 59, 58, 51, and 35). It should be noted that two earlier studies in Ghana showed that HPV 59 was one of the two highest HPV types detected in tissues obtained from pathologically diagnosed invasive cervical cancers [10, 23]. Furthermore, although cost-effectiveness analysis are needed [38] to take a firm decision on which sample collection methods to use for a population-based HPV screening, and which type of vaccine to use in Ghana, it is undoubtedly clear that the HPV infection data obtainable with self-collected specimens, (which detected the high-risk HPV 33, that was not detected by PC specimens) will be a better means for assessing pre and postHPV vaccination benefits, in Ghana.

The overall HPV prevalence obtained with PC specimens (23.3%) was lower compared to those reported for Benin, 33.2% [39]; Burkina-Faso, 54.0% among a subgroup of HIV negative women [40]; Nigeria, 26.3% [41]; 31.1% in Côte d'Ivoire, among a population control [42] and 33.3% among pregnant women in Ghana [43]. However, it was higher than that for a rural Gambian community, 13.4% [44]. Furthermore, this prevalence is similar to that reported (21.5%) in a meta-analysis of studies conducted with data from Senegal, Nigeria, Guinea, Gambia, and Cote d'Ivoire [45]. Therefore, the result of this study adds to the growing evidence of geographical differences in HPV prevalence among countries of the West African region. It must, however, be noted that such differences in prevalences are also influenced by differences in the study design, category and age range of women, methods of HPV detection and genotyping, and the HIV status of the participant [46, 47].

The higher overall HPV prevalences with self-collected specimens (Tables 4 and 5) were similar to the pattern of differences reported in a study of South African adolescents [48], which are explainable by the fact that self-collection were more likely to have sampled the vulva, vagina, and the cervix, as compared to only the cervix by the provider-collection of specimens [49]. Other contributing factors may include, the fact that low-risk HPV types are more associated with the vaginal epithelium [49, 50]. On the other hand, the higher prevalence of high-risk HPV with SC sample is contrary to the fact that high-risk HPVs differentially associate with the cervical and vaginal epithelia [51], however, optimal performance of self-collection and the extent of exfoliation of the cervical epithelium into the vagina may contribute to the higher high-risk HPV prevalence with SC samples [17, 49, 52, 53].

Although , a review had shown that a wide range of levels of agreement (*k* = 0.24 − 0.96) have been reported between SC and PC, for both overall HPV and high-risk HPV detection [51, 54], the Kappa analysis in this study, showed a moderate level of agreement between the two methods (*k* = 0.402, *k* = 0.321 and 0.328). Additionally, the high level of concordance (77.9%) between the two methods, in detecting high-risk (HR) HPVs and the moderate level of concordance (67.3%) for overall HPV (Table 5), are not uncommon. Other studies have reported high concordances (>75.0%) for overall HPV infections either with PC performing better [17, 53], or SC performing better [16]. These imply that the use of self-collection in Ghana for cervical cancer screening is more like to be more informative than provider-collection concerning HPV infection. The detection of HPV 33, 53, 70, 72, and 90 by SC specimens and not PC specimens is critical particularly in respect of the high-risk type HPV33. This information should make a difference in preference for the nonavalent vaccine.

Although this study was specifically powered to determine HPV prevalences, the study compared the associations of the known risk factors with HPV positivity (overall, high-risk, and multiple HPV infections) obtained with SC and with PC specimens. The directionalities (OR of <1 or >1) of the crude odds ratios determined with SC specimens were often as expected, as has been reported by studies and reviews of similar studies [41, 55–59]. However, for the crude odds ratios determined with PC specimens, the directionalities were mostly not as expected. Additional data present in Tables 1 and 2, specifically, those for which there were significant differences in participant reporting, indicate the characteristics that may influence the attendance of women for cervical cancer screening; theses should, therefore, be considered in planning such activities.

With these high HPV infections prevalences, it was expected that between 2.7% and 20.0% of low-grade cervical lesions would have been detected, as has been reported in some studies in West African countries with similar sample sizes [39, 40, 46, 60]; however, no cervical lesion was detected. Aside issues relating to the quality of Pap smear testing, the small sample size and the undetermined extent of HPV persistence, the nondetection of a cervical lesion may be explainable in part with data on genetic variants of the high-risk HPV genotypes detected in this study [61–63].

In respect to the Pap smear analysis (Table 7), the reasons for the high unsatisfactory smears were not clear, and there was no statistically significant association between the adequacy of the smear and the location of specimen collection and smear preparation.

## 5. Conclusion

The prevaccination genital HPV infections burden among women in the Akuse subdistrict, as determined with self-collected specimens, indicated higher vaccine-type HPVs, and high-risk HPV prevalences when compared to the burden determined with provider-collected specimens. Additionally, there were multiple HPV infections which included high-risk HPV infections that are not preventable by the available vaccines, neither directly nor by cross-protection. This implies that even with a successful implementation HPV vaccination if introduced in Ghana, there is still the need to continue with the screening of women. It is therefore strongly recommended to the Ministry of Health, Ghana that self-collection (SC) and HPV testing be considered for a national cervical cancer screening programme in Ghana, particularly for the assessment of the impact of HPV vaccination.

## Figures and Tables

**Table 1:** Distribution of the demographic characteristics of women who did or did not report for specimen collection.

Variables	Categories	^#^Number (%) of women who			Pearson chi-square (*p*-value)
		Reported	Did not report	Total	
Age (categorised)	15–19	12 (4.8)	14 (8.7)	26 (6.3)	10.806 (0.289)
	20–24	36 (14.3)	29 (18.0)	65 (15.8)	
	25–29	61(24.3)	36 (22.4)	97 (23.5)	
	30–34	27 (10.8)	19 (11.8)	46 (11.2)	
	35–39	36 (14.3)	16 (9.9)	52 (12.6)	
	40–44	32 (12.7)	19 (11.8)	51 (12.4)	
	45–49	14 (5.6)	16 (9.9)	30 (7.3)	
	50–54	16 (6.4)	6 (3.7)	22 (5.3)	
	55–59	9 (3.6)	4 (2.5)	13 (3.2)	
	60 or older	8 (3.2)	2 (1.2)	10 (2.4)	
	Total	251	161	412	

Religion	Christian	242 (96.4)	131 (81.4)	373 (90.5)	26.91 (≤0.001)^∗^
	Muslim	9 (3.6)	26 (16.1)	35 (8.5)	
	Other	0 (0.0)	4 (2.5)	4 (1.0)	
	Total	251	161	412	

Marital status	Unmarried	79 (32.0)	71 (44.4)	150 (36.9)	6.406 (0.011)^∗^
	Married	168 (68.0)	89 (55.6)	257 (63.1)	
	Total	247	160	407	

Educational status	No formal education	40 (16.1)	28 (17.5)	68 (16.6)	6.035 (0.197)
	Primary	51 (20.5)	26 (16.3)	77 (18.8)	
	Junior secondary	112 (45.0)	82 (51.3)	194 (47.4)	
	Senior secondary	28 (11.2)	20 (12.5)	48 (11.7)	
	Post-secondary	18 (7.2)	4 (2.5)	22 (5.4)	
	Total	249	160	409	

Occupation	Unemployed	19 (7.8)	6 (3.8)	25 (6.2)	7.320 (0.120)
	Formal employment	40 (16.4)	26 (16.5)	66 (16.4)	
	Skilled worker	62 (25.4)	31 (19.6)	93 (23.1)	
	Trader	101(41.4)	71 (44.9)	172 (42.8)	
	Agro-worker	22 (9.0)	24 (15.2)	46 (11.4)	
	Total	244	158	402	

^∗^Significant difference within the categories. ^#^The total number of women who completed the questionnaire was 415.

**Table 2:** Difference in sexual/reproductive characteristics of women who did or did not report for specimen collection.

Variables	Categories	^#^Number (%) of women who (*N* = 415)			Pearson chi-square (*p*-value)
		Reported	Did not report	Total	
Age at first sexual intercourse (categorised)	<20	155 (62.8)	95 (63.3)	250 (63.0)	0.013 (0.908)
	≥20	92 (37.2)	55 (36.7)	147 (37.0)	
	Total	247	150	397	

Lifetime number of sexual partners (categorised)	1	52 (21.1)	39 (26.0)	91 (22.9)	2.387 (0.303)
	2	75 (30.4)	36 (24.0)	111 (28.0)	
	≥3	120 (48.6)	75 (50.0)	195 (49.1)	
	Total	247	150	397	

Use of condom during sexual intercourse	No	148 (59.2)	92 (57.9)	240 (58.7)	0.072 (0.789)
	Yes	102 (40.8)	67 (42.1)	169 (41.3)	
	Total	250	159	409	

Number of abortion (categorised)	1	73 (62.4)	28 (50.0)	101 (58.4)	2.394 (0.122)
	≥2	44 (37.6)	28 (50.0)	72 (41.6)	
	Total	117	56	173	

Number of pregnancies (categorised)	1	34 (15.3)	23 (17.3)	57 (16.1)	0.521 (0.771)
	2–4	100 (45.0)	62 (46.6)	162 (45.6)	
	≥5	88 (39.6)	48 (36.1)	136 (38.3)	
	Total	222	133	355	

Number of miscarriages (categorised)	1	42 (63.6)	25 (62.5)	67 (63.2)	0.014 (0.906)
	≥2	24 (36.4)	15 (37.5)	39 (36.8)	
	Total	66	40	106	

Duration of OC use	<1 year	33 (56.9)	14 (43.8)	47 (52.2)	7.823 (0.020)^∗^
	1–4 years	18 (31.0)	18 (56.3)	36 (40.0)	
	>4 years	7 (12.1)	0 (0.0)	7 (7.8)	
	Total	58	32	90	

Sexual age (categorised)	<11	105 (42.5)	70 (47.0)	175 (44.2)	1.317 (0.725)
	11–19	62 (25.1)	35 (23.5)	97 (24.5)	
	20–29	51 (20.6)	31 (20.8)	82 (20.7)	
	>29	29 (11.7)	13 (8.70)	42 (10.60)	
	Total	247	149	396	

Male sexual partner	None currently	33 (13.1)	28 (17.6)	61 (14.9)	1.531 (0.216)
	Currently have	218 (86.9)	131 (82.4)	349 (85.1)	
	Total	251	159	410	

Abortion	Not aborted	133 (53.2)	105 (65.2)	238 (57.9)	5.802 (0.016)^∗^
	Have aborted	117 (46.8)	56 (34.8)	173 (42.1)	
	Total	250	161	411	

^∗^Significant difference within the categories. ^#^The total number of women who completed the questionnaire was 415.

**Table 3:** HPV genotype prevalence determined with each type of specimen.

HPV genotype	Self-collected specimens, *n* = 244				Provider-collected specimens, *n* = 230			
	Total infections	Multiple infection	Prevalence	95% CI^∗^	Total infections	Multiple infection	Prevalence	95% CI^∗^
HPV16^b, q, n^	15	9	5.9	3.0–9.0	6	1	2.4	1.0–5.0
HPV35	12	9	4.7	2.0–8.0	7	2	2.8	1.0–5.0
HPV40	12	9	4.7	2.0–8.0	4	4	1.6	0.0–4.0
HPV45^n^	11	7	4.3	2.0–7.0	5	2	2.0	0.0–4.0
HPV58^n^	10	5	4.0	2.0–7.0	7	1	2.8	1.0–5.0
HPV18^b, q, n^	9	7	3.6	1.0–6.0	6	1	2.4	1.0–5.0
HPV66	8	5	3.2	1.0–6.0	6	6	2.4	1.0–5.0
HPV59	7	5	2.8	1.0–5.0	3	2	1.2	0.0–3.0
HPV6^q, n^	7	4	2.8	1.0–5.0	3	0	1.2	0.0–3.0
HPV31^n^	7	2	2.8	1.0–5.0	2	0	0.8	0.0–2.0
HPV74	7	2	2.8	1.0–5.0	1	0	0.4	0.0–1.0
HPV42	5	3	2.0	0.0–4.0	5	4	2.0	0.0–4.0
HPV52^n^	5	3	2.0	0.0–4.0	4	2	1.6	0.0–4.0
HPV51	5	4	2.0	0.0–4.0	3	3	1.2	0.0–3.0
HPV62	5	3	2.0	0.0–4.0	2	1	0.8	0.0–2.0
HPV81	5	3	2.0	0.0–4.0	1	1	0.4	0.0–1.0
HPV83	5	4	2.0	0.0–4.0	1	0	0.4	0.0–1.0
HPV72	5	5	2.0	0.0–4.0	ND	ND	ND	
HPV90	5	4	2.0	0.0–4.0	ND	ND	ND	
HPV67	4	2	1.6	0.0–3.0	1	1	0.4	0.0–1.0
HPV53	4	2	1.6	0.0–3.0	ND	ND	ND	
HPV56	3	3	1.2	0.0–3.0	1	0	0.4	0.0–1.0
HPV33^n^	3	3	1.2	0.0–3.0	ND	ND	ND	
HPV70	3	3	1.2	0.0–3.0	ND	ND	ND	
HPV87	2	2	0.8	0.0–2.0	2	1	0.8	0.0–2.0
HPV39	2	2	0.8	0.0–2.0	1	0	0.4	0.0–1.0
HPV30	2	1	0.8	0.0–2.0	1	1	0.4	0.0–1.0
HPV54	2	2	0.8	0.0–2.0	1	0	0.4	0.0–1.0
HPV44	2	1	0.8	0.0–2.0	ND	ND	ND	
HPV86	2	0	0.8	0.0–2.0	ND	ND	ND	
HPV82	1	0	0.4	0.0–1.0	1	0	0.4	0.0–1.0
HPV84	1	1	0.4	0.0–1.0	1	0	0.4	0.0–1.0
HPV68	1	1	0.4	0.0–1.0	ND	ND	ND	
HPV69	1	1	0.4	0.0–1.0	ND	ND	ND	
HPV85	1	1	0.4	0.0–1.0	ND	ND	ND	
HPV91	1	0	0.4	0.0–1.0	ND	ND	ND	
HPV73	1	0	0.4	0.0–1.0	ND	ND	ND	
HPV43	ND	ND	ND		1	0	0.4	0.0–1.0

ND = not detected ^∗^Differences in the prevalences were not significant since each pair of 95% CI overlapped. ^b^Bivalent HPV type, ^q^quadrivalent HPV type, ^n^nonavalent HPV type.

**Table 4:** HPV prevalences, proportions, and the extent of agreements between self and provider-collected specimens.

HPV type	Self-collection	Provider-collection	Chi-square (≤*p*-value )	Cohen's Kappa, (*p*-value)
	Prevalence (95% CI)	Prevalence (95% CI)		
Overall HPVs	43.1 (38.0–51.0)	23.3 (19.0–31.0)	0.001	0.321 (0.0001)
High-risk HPVs	27.2 (23.0–34.0)	16.6 (14.0–24.0)	0.001	0.402 (0.0001)
Low-risk HPVs	23.3 (19.0–30.0)	9.9 (6.0–14.0)	0.001	0.328 (0.0001)

Probable high-risk HPVs	4.7 (2.0–8.0)	0.4 (0.0–1.0)	0.001	0.147 (0.0001)
BVT HPV's	5.9 (3.0–9.0)	0.9 (0.14–2.84)	0.001	
QVT HPVs	12.7 (8.95–17.3)	6.5 (3.54–10.3)	0.001	
NVT HPVs	27.5 (22.1–33.3)	14.3 (10.3–19.3)	0.001	

*Infection categories*	*n*, (Proportion)^a^	*n*, (Proportion)^b^		
Single infection^c^	62 (56.9%)	43 (72.9%)	0.001	
Multiple infections^c^	49 (43.1%)	16 (27.1%)	0.001	
Multiple infections involving BVT HPVs only^c^	1 (0.92%)	0 (0.0%)	—	
Multiple infections involving QVT HPVs only^c^	1 (0.92%)	0 (0.0%)	—	
Multiple infections involving NVT HPVs only^c^	4 (3.67%)^f^	0 (0.0%)^g^	—	
Multiple infections involving any BVT and nonBVT HR HPVs^c^	7 (6.427%)	0 (0.0%)	—	
Multiple infections involving any QVT and nonQVT HR HPVs^c^	10 (9.17%)^ d^	1 (1.69%)^e^	0.001	
Multiple infections involving any QVT and nonQVT LR HPVs^c^	8 (7.34%)	1 (1.969%)	0.001	
Multiple infections involving any NVT and nonNVT HR HPVs^c^	12 (11.00%)	3 (5.08%)	0.001	
Multiple infections involving any NVT and nonNVT LR HPV(s)^c^	16 (14.68%)	4 (6.77%)	0.001	

BVT = Bivalent vaccine-type, QVT = quadrivalent vaccine-type, NVT = nonavalent vaccine-type. Prevalence was determined with 244 SC specimens and 230 PC specimens. Chi-square and Kappa analysis were performed with only the 266 pairs of SC and PC specimens. ^c^Single and multiple infections were reported as proportions ^a^of the 109 HPV positive with SC specimens; ^b^ of the 59 HPV positive with PC specimens. ^d^ The other high-risk HPVs were HPV59, HPV45, HPV35, HPV51, HPV58; ^e^ the other high-risk HPV were HPV59; ^f^ the other high-risk HPVs were HPV59, HPV35, HPV51, HPV39; ^g^ the other high-risk HPVs were HPV51.

**Table 5:** Concordance and discordance in the detection of HPV with self and provider-collected specimens.

HPV status	Self-collected specimens	Provider-collected specimens			McNemar's test (*p*-value)
		Negative	Positive	Total	
Overall HPVs	Negative	108 (47.8%)	13 (5.8%)	121	*≤*0.001
	Positive	61 (27.0%)	44 (19.5%)	105	
	Total	169	57	226	
HR HPVs	Negative	147 (65.2%)	13 (5.7%)	160	*≤*0.001
	Positive	37 (16.3%)	29 (12.8%)	66	
	Total	184	42	226	
LR HPVs	Negative	166 (73.5%)	6 (2.7%)	172	*≤*0.001
	Positive	39 (17.3%)	15 (6.6%)	54	
	Total	205	21	226	

% are of the overall total of participants who performed both methods, *n* = 226.

**Table 6:** Crude odds ratios for the associations between sexual characteristics and positive HPV infections status.

Sexual characteristics	Category	Crude Odds ratio (95% CI), with					
		Self-collected specimens			Provider-collected specimens		
		Overall HPV	HR HPV	Multiple HPV	Overall HPV	HR HPV	Multiple HPV
Age (years)		0.96 (0.94–0.99)	0.96 (0.94–0.99)	0.97 (0.94–1.00)	0.99 (0.97–1.02)	1.00 (0.97–1.03)	0.97 (0.92–1.03)
Age at first sexual intercourse (years)	<20		Reference			Reference	
	≥20	0.64 (0.37–1.09)	0.46 (0.24–0.85)^∗^	0.60 (0.29–1.22)	1.15 (0.62–2.11)	1.16 (0.59–2.31)	1.05 (0.36–3.01)
Currently have a male sexual partner	No		Reference			Reference	
	Yes	0.80 (0.38–1.67)	0.62 (0.29–1.35)	0.67 (0.27–1.68)	0.64 (0.29–1.41)	0.79 (0.32–1.96)	2.19 (0.28–17.37)
Lifetime number of sexual partners		1.04 (0.87–1.25)	1.07 (0.88–1.30)	1.14 (0.90–1.45)	1.01 (0.83–1.25)	1.03 (0.82–1.29)	1.12 (0.81–1.55)
Sexual-age (years)^	<11		Reference			Reference	
	11–19	0.35 (0.18–0.67)^∗^	0.36 (0.17–0.76)^∗^	0.22 (0.08–0.58)^∗^	0.49 (0.22–1.07)	0.62 (0.27–1.46)	0.25 (0.05–1.18)
	20–29	0.31 (0.15–0.63)^∗^	0.38 (0.17–0.84)^∗^	0.16 (0.05–0.50)^∗^	0.38 (0.15–0.95)^∗^	0.33 (0.11–1.02)	0.15 (0.02–1.24)
	>29	0.54 (0.23–1.27)	0.65 (0.26–1.63)	0.75 (0.28–2.01)	1.13 (0.45–2.81)	1.27 (0.47–3.42)	0.71 (0.14–3.49)
Contracted STI	No		Reference		Reference		
	Yes	1.33 (0.79–2.22)	1.34 (0.76–2.3)	1.45 (0.73–2.86)	1.14 (0.63–2.08)	1.15 (0.58–2.27)	0.78 (0.28–2.18)
Condom use	No		Reference			Reference	
	Yes	1.44 (0.86–2.41)	1.09 (0.62–1.92)	2.03 (1.04–3.98)^∗^	1.15 (0.63–2.09)	1.08 (0.55–2.12)	1.87 (0.67–5.27)

^∗^The odds of the association was significant, since the range of 95% CI excludes 1.0. Multivariable analysis (including either the crude significant characteristics or all the characteristics) indicated that all the characteristics were not significantly associated with any of the categories of HPV positivity status, for each of the collection methods (SC and PC). ^ Sexual age is the numbers of years since a woman had her first sexual intercourse.

**Table 7:** Distribution of diagnosis based on Pap smear evaluation.

Diagnosis	Frequency	Percentage
Dyskaryosis	0	0.0
Acute vaginitis	4	2.1
Atrophic cervicitis	9	4.2
Bacterial vaginosis	15	7.4
Candida infection	13	6.3
No abnormality detected	170	80.0

Total	212	100.0

## Data Availability

The datasets used and/or analysed during the current study are available from the corresponding author on reasonable request.

## Abbreviations

HPV: Human papillomavirus
SC: Self-collected/collection
PC: Provider-collected/collection
EDTA: Ethylenediaminetetraacetic acid
GHS: Ghana health service,
ERC: Ethical review committee
PE: Streptavidin-phycoerythrin
TMAC: 1-Tetramethyl ammonium chloride
CI: Confidence interval
OR: Odds ratio.
